# Medication adherence halves COPD patients’ hospitalization risk – evidence from Swiss health insurance data

**DOI:** 10.1038/s41533-024-00361-2

**Published:** 2024-03-07

**Authors:** Anja Y. Bischof, Johannes Cordier, Justus Vogel, Alexander Geissler

**Affiliations:** School of Medicine, University of St. Gallen, St. Gallen, Switzerland

**Keywords:** Chronic obstructive pulmonary disease, Health policy

## Abstract

Medication adherence is vital for patients suffering from Chronic Obstructive Pulmonary Disease (COPD) to mitigate long-term consequences. The impact of poor medication adherence on inferior outcomes like exacerbations leading to hospital admissions is yet to be studied using real-world data. Using Swiss claims data from 2015-2020, we group patients into five categories according to their medication possession ratio. By employing a logistic regression, we quantify each category’s average treatment effect of the medication possession ratio on hospitalized exacerbations. 13,557 COPD patients are included in the analysis. Patients with high medication adherence (daily medication reserve of 80% to 100%) are 51% less likely to incur exacerbation following a hospital stay than patients with the lowest medication adherence (daily medication reserve of 0% to 20%). The study shows that medication adherence varies strongly among Swiss COPD patients. Furthermore, high medication adherence immensely decreases the risk of hospitalized exacerbations.

## Introduction

In 2019, Chronic Obstructive Pulmonary Disease (COPD) was the third leading cause of death worldwide^1^. Typical COPD symptoms are shortness of breath, sputum, and cough^2^. In Switzerland, about 400,000 people suffer from COPD^3^. Annual direct healthcare expenditures are CHF 603-847 million, while the indirect costs of COPD amount up to CHF 932 million per year^4^. The prevalence of COPD is particularly high in individuals with low socioeconomic status^5^.

To treat COPD sustainably and slowing down the progress of the disease, COPD patients are prescribed long- and short-acting medication. Long-acting medication should be taken regularly to reach its full effect. Thus, it differs from short-acting medication, which is taken according to acute need such as during shortness of breath^2,6^. Taking long-acting medication as prescribed alleviates disease-specific symptoms, slows disease progression, and prevents hospitalization due to acute exacerbation^7^. Acute exacerbations are especially harmful as they result in additional therapy, imply a setback in COPD patients’ health-related quality of life, raise the risk of iatrogenic harm, and diminish lung function long-term^8,9^.

High medication adherence can help to better control disease progression, reduce hospitalizations due to acute exacerbations and ultimately reduce cost^7^. According to the WHO, medication adherence refers to “the extent to which the person’s behavior corresponds with the agreed recommendations from a healthcare provider”^10^. A globally conducted study concluded that in clinical trials medication adherence reaches up to 80%^11^, while in real world settings, based on US data, medication adherence is solely around 30%^12^. Similarly, a Korean study showed that adherence is low by analyzing a nationwide health insurance dataset. The percentage of patients with high adherence was only around 34.7% and declined to 22.3% over 4 years^13^.

Implementing high medication adherence in COPD patients depends on, among others, patients’ beliefs about medication and concerns about potential side effects, experiences and satisfaction with medication effectiveness, personal circumstances, and health status^14^. Moreover, the patient-physician relationship and the physician’s availability are also relevant factors for a high medication adherence^15–18^. Further, COPD patients report personal challenges related to medication adherence, e.g., understanding appropriate intake or adhering to the correct timing of intake^18^.

Some studies have already investigated the relation of medication adherence and acute exacerbations^13,19,20^. However, only a few studies focused on the relationship between medication adherence and hospitalization caused by acute exacerbations (see exemplarily Suh et al. ^21^, Chen et al. ^22^, or Weir et al. ^23^). Nevertheless, the medication adherence of COPD patients in Switzerland is unknown.

We use Swiss health insurance data to investigate medication adherence of Swiss COPD patients and to analyze the effects of low medication adherence on the risk of hospitalization. Hence, our research questions are:

- How is medication adherence distributed among COPD patients?
- How does inadequate medication adherence impact the likelihood for a hospitalized exacerbation?

We aim to use these results to contribute to the ongoing discussion regarding strategies for improving medication adherence among individuals with this chronic condition. Given the substantial prevalence of this patient group, even minor adjustments could potentially yield significant benefits. Similar to previous studies^19–21^, we estimate medication adherence from the proportion of days covered (PDC), assuming that COPD patients had enough medication at home. We investigate whether low adherence to prescribed long-acting medications made exacerbations more likely using a logistic regression. Thus, we categorize COPD patients into categories according to their PDC.

## Results

### Descriptive results

**Fig. 1:**
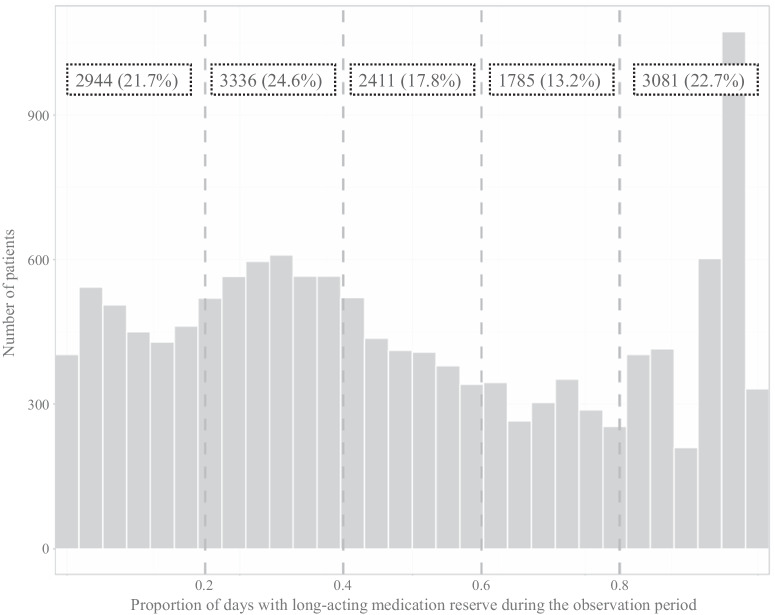
Grouping of COPD patients according to the proportion of days covered (PDC). The *X*-axis shows the proportion of days covered (PDC) over all days in the observation period and the *Y*-axis plots the number of COPD patients. Categories are indicated with dashed lines. The number of COPD patients per category and their share of the total sample are indicated in the rectangles at the top.

Figure 1 shows the distribution diagram of the number of COPD patients by PDC. Category 1 on the left, for instance, includes all COPD patients who had a positive medication reserve on 0 to 20% of the days of the observation period.

46.3% of the COPD patients are grouped in the first two categories, i.e., about half of the patients has a PDC of less than 40% of the days during the observation period. At the same time, the number of COPD patients in categories 3 and 4 is relatively low and second highest in category 5, i.e., the proportion of COPD patients who have a rather high PDC.

Of the 13,557 COPD patients, 1721 (12.6%) were hospitalized due to an exacerbation. The lowest rate of exacerbations is present in category 5 with 225 out of 3081 (7.2%) COPD patients, whereas the first category includes the highest rate of exacerbations with 619 out of 2944 (21.0%) COPD patients (see Table 1). Accordingly, the prescription of short-acting medications – provided during an acute worsening of the lung function – is highest in category 1. Regarding age, COPD patients in category 5 are on average ~3.5 years younger than in category 1. Furthermore, the rate of premium reduction is ~40% for all categories, whereas COPD patients in category 1 most often receive premium reduction with about 42.8%, meaning they have the lowest incomes.

**Table 1:** Number of COPD patients, number and proportion of exacerbations, and mean of the model variables per category.

	Category 1	Category 2	Category 3	Category 4	Category 5	Total
Number of observations (share of total) (%)	2944 (21.7)	3336 (24.6)	2411 (17.8)	1785 (13.2)	3081 (22.7)	13,557 (100)
Number (share of category) exacerbations (%)^a^	619 (21.0)	446 (13.4)	268 (11.1)	163 (9.1)	225 (7.2)	1721 (12.6)
Average age in years (standard deviation)	68.45 (10.64)	66.51 (11.12)	66.05 (11.27)	66.08 (11.36)	64.86 (11.30)	66.42 (11.18)
Premium reduction (%)	42.8	41.6	40.0	40.1	38.6	40.7
Increase of the deductible (%)	2.0	2.6	2.6	2.6	2.4	2.4
Reduction of the deductible (%)	5.5	7.6	6.7	4.4	5.3	6.0
Hospitalized exacerbation during the observation period (%)^b^	11.2	5.5	4.7	3.3	1.4	5.4
Purchase of short-acting medication (%)	28.3	20.8	19.0	17.1	12.5	19.7
Purchase of methylxanthine (%)	0.3	0.2	0.3	0.1	0.1	0.2
Purchase of phosphodiesterase 4 inhibitors (%)	2.3	1.4	0.7	0.5	0.1	1.1
Purchase of of mucolytic (%)	9.5	9.0	8.9	8.6	8.6	9.0

^a^This variable indicates the total number and the corresponding share of a category of all exacerbations between 2015 and 2020

^b^This variable relates to the exacerbations which have taken place during the two-year observation period of individuals belonging to the different categories.

### Regression results: Impact on the exacerbation likelihood

Regarding the results of the main model, the regression analysis showed a significant effect between higher medication adherence and hospitalized exacerbations (see Table 2). Control variables with significant associations to hospitalized exacerbations were premium reduction, age, prescription of short-acting medications, and prescription of phosphodiesterase-4 inhibitors.

**Table 2:** Regression output on the association of higher medication adherence with hospitalized exacerbations.

Variable	Abbreviation	Estimate coefficient	Std. error	OR	95% CI	Significance marker
Category 2 of the proportion of days with medication reserve	Q2	−0.319	0.074	0.73	(0.63–0.84)	***
Category 3 of the proportion of days with medication reserve	Q3	−0.479	0.085	0.62	(0.52–0.73)	***
Category 4 of the proportion of days with medication reserve	Q4	−0.637	0.100	0.53	(0.43–0.65)	***
Category 5 of the proportion of days with medication reserve	Q5	−0.709	0.089	0.49	(0.41–0.59)	***
Current deductible = 500	F_500_	−0.018	0.065	0.98	(0.86–1.12)	–
Current deductible = 1000	F_1000_	−0.115	0.186	0.89	(0.61–1.29)	–
Current deductible = 1500	F_1500_	0.238	0.140	1.27	(0.96–1.68)	–
Current deductible = 2000	F_2000_	0.024	0.371	1.02	(0.49–2.15)	–
Current deductible = 2500	F_2500_	−0.043	0.236	0.96	(0.60–1.54)	–
Premium reduction	R	0.261	0.057	1.30	(1.16–1.45)	***
Age	A	0.017	0.002	1.02	(1.01–1.02)	***
Increase of the franchise	F+	−0.138	0.237	0.87	(0.54–1.40)	–
Reduction of the franchise	F-	−0.181	0.138	0.83	(0.63–1.10)	–
Hospitalized exacerbation during the observation period	E_t-2_	1.580	0.087	4.85	(4.08–5.78)	***
Prescription of short-acting medication	B	1.280	0.058	3.60	(3.20–4.04)	***
Methylxanthine prescription	M	0.510	0.476	1.67	(0.64–4.31)	–
Prescription of phosphodiesterase 4 inhibitors.	P	1.410	0.194	4.10	(2.78–6.04)	***
Prescription of mucolytic	K	−0.186	0.100	0.83	(0.68–1.01)	–
Intercept	${\beta }_{0}$	31.600	5.020	–	(0.63–0.84)	***

The significance levels were set at *p < 0.1, **p < 0.05, and ***p < 0.01; OR = Odds ratio; CI = Confidence Interval

*OR* odds ratio; *CI* confidence interval

**Fig. 2:**
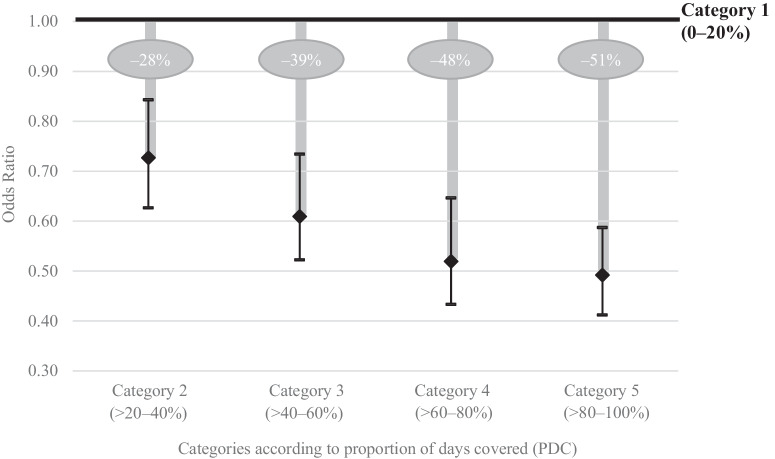
Hospitalization likelihood due to exacerbations in relation with higher medication adherence. The figure illustrates the decrease in the likelihood of hospitalized exacerbations per category. Category 1 functions as the reference for the comparison with categories 2 to 5. The whiskers display the 95% confidence intervals.

Figure 2 shows a large effect of higher medication adherence on the probability of exacerbation, using category 1 as the reference value for each of the categories 2–5. If the odds ratio of a category is less than 1, this means that COPD patients in this category are less likely to be hospitalized due to an exacerbation during the observation period compared to category 1. For example, Fig. 2 displays that the probability of an exacerbation is already about 28% lower if the PDC is >20-40% (category 2). COPD patients with a PDC of more than 80% (category 5) have a 51% lower probability of being hospitalized due to an exacerbation. We observe a decreasing marginal utility of continuous medication reserve (i.e., belonging to a higher category). Further, each category’s whisker stands for the confidence interval. The confidence intervals decrease for higher medication adherence – indicating that a higher medication adherence allows for more accurate estimates.

## Discussion

We contribute to the literature in 3 ways: (1) We show that medication adherence – measured by the PDC – varies substantially among COPD patients; (2) high medication adherence potentially halves the risk of hospitalized exacerbations; and (3) utilizing real-world (insurance) data yields accurate figures for medication adherence.

We grouped the PDC of COPD patients into five categories. With regard to the distribution, categories 1 and 2 include 6280 COPD patients (48% of total sample). Thus, a large number of COPD patients have a PDC of less than 40%. This finding is critical because it implies that almost half of COPD patients fail to take their medication on four out of seven days. Lastly, almost one fourth of our sample falls into category 5. This finding supports the literature as in clinical practice only around 30% of COPD patients are found to be adherent to their medication prescriptions^12^.

When comparing the characteristics of the patients grouped within the category, especially the share of hospitalized exacerbations, prescription of short-acting medication, and the number of premium reductions are striking. The lowest rate of exacerbations is present in category 5 with 225 out of 3081 (7%) COPD patients, whereas the first category includes the highest rate of exacerbations with 619 out of 2944 (21%) COPD patients. This relation states a first indication for an association of PDC and hospitalized exacerbations. Additionally, the rate of short-acting medication is highest in category 1 with 28%. Comparing this share with categories 4 and 5, the prescription rate is clearly lower with only 12% in category 4 and 19% in category 5. Short-acting medications are prescribed in case of an acute worsening of the disease symptoms to prevent or treat an exacerbation^2^. Therefore, the share of prescribed short-acting medication further supports the assumption that COPD patients in category 1, i.e., with low medication adherence, are more likely to suffer from exacerbations.

Furthermore, the share of premium reduction recipients among the COPD patients is remarkably high. Premium reductions are granted to people living in modest economic circumstances, where the family and financial situation is decisive for receiving premium reductions^24^. In our sample, around 39% to almost 43% of all COPD patients – depending on the corresponding category – receive premium reduction. Compared to the average Swiss population, approximately 28% of all insured persons received a premium reduction in 2020^25^. The higher share of premium reduction recepients is a sign for lower socioeconomic status among COPD patients – as already highlighted by Eisner et al. ^5^.

In addition, potential influencing factors on hospitalized exacerbations were analyzed. First, higher medication adherence lowers the risk for acute exacerbations, which is in line with the literature^21–23,26^. Our analysis shows that the risk for hospitalized exacerbations can be decreased ~51% in case of high adherence (category 5) compared to non- respectively low adherence (category 1). However, also COPD patients in categories 2 to 4 benefit from lower risks for exacerbations (between 28%-48% less likelihood) compared to COPD patients belonging to category 1. Similar results were also retrieved in RCTs where, for example, Vestbo et al. ^11^. showed that adherence to inhaled medication is significantly associated with reduced risk of death and admission to hospital due to exacerbations in COPD. Furthermore, Torres-Robles et al. ^27^. found that medication adherence improves clinical outcomes in COPD patients and that interventions on increasing medication adherence show positive results. These insights shows that COPD patients would already benefit if general practitioners, outpatient specialists, and other medical professionals could manage supporting patients in moderately increasing their medication adherence. Potential approaches for increasing COPD patients’ medication adherence are individualized educational interventions^28,29^, reminders, motivational strategies, shared decision making or direct feedback on medication use^29^. These approaches could also be incorporated into digital health interventions (for example, Spielmanns et al.^30^).

Medication adherence is categorized into three main stages: initiation, implementation, and discontinuation^31^. The focus of this study is on the intersection of the two latter stages – implementation and discontinuation – by calculating the PDC. Although most COPD patients do not take their medications as prescribed (i.e., no full implementation), they do also not completely quit taking their medications (i.e., no full discontinutation). This inconsistent behavior of COPD patients leads to the assumption that they face certain challenges in their adherence habit such as personal beliefs about the effectiveness of the prescribed medication, patient-physician relationship, or insecurities in the right usage of the medication^14–17^. Rising awareness of these challenges in medication adherence plus knowing the factors (such as higher age, receiving premium reduction, or prior exacerbations) being significantly associated to a higher risk of exacerbations, health policy initiatives should sustainably strengthen COPD patients’ medication adherence, e.g. when implementing chronic care programs and/or digital health assistants.

The limitations of this study are twofold: firstly, due to the missing patient diagnosis in the dataset, the number of COPD patients included in the dataset are approximated with two inclusion criteria: taking prescribed long-acting medication and age over 40 years. This means that, on the one hand, we might underestimate the number of COPD patients as patients might not take any medication yet or they stopped taking medication. On the other hand, we might include asthma patients in our sample because there might be asthma patients older than 40 years taking long-acting prescriptions. Both issues should be marginal, however, and not strongly influence our results. Moreover, the advantage of claims data enabling us to observe patients intersectorally is more essential than the limitation of potentially underestimating the number of COPD patients or including single patients with a different diagnosis.

Secondly, due to the empirical setting of this study, the number of observed variables is limited. Two areas with potentially unobserved control variables include the COPD patient’s personal motivation and financial situation. Personal motivation is difficult to quantitatively replicate based on the used dataset but may have an impact on both medication reserve and the likelihood of exacerbation. Furthermore, high personal motivation might positively influence COPD patients’ health literacy and their body awareness. These two factors potentially reduce the exacerbation risk. However, in our study, we only focus on the association of the PDC with exacerbation likelihood. Therefore, we might overestimate this effect as we neglect the association of motivation with exacerbation likelihood. Additionally, the financial situation may influence the utilization of healthcare services, as there is some cost sharing in the Swiss insurance system between insured persons and insurances. We aimed to consider this by including premium reduction as control variable. However, it is only a proxy, since the variable premium reduction only adjusts for lower income groups. If there is no premium reduction, the exact income level is still unknown.

For future research, a better understanding of COPD patients’ needs on potential supporting mechanisms to promote higher medication adherence has to be established. Through a deepened understanding of current needs or challenges faced by COPD patients, researchers may be able to develop supportive tools to empower COPD patients in their daily lives to deal with the challenges of COPD and to increase their medication adherence. On the one hand, the risk of hospitalized exacerbations can be decreased, whereas on the other hand, the COPD patient’s health-related quality of life should be conserved through a potential slowdown of the progression of the disease.

To conclude, medication adherence according to prescription halves the risk for hospitalized exacerbations. Generally, medication adherence is low meaning that there is vast potential to improve patient-relevant outcomes without “inventing” a new treatment but by supporting COPD patients. There are two main levers to do this: (1) Strengthening health literacy so that patients understand the importance of regular medication intake and (2) structured, physician-led support programs such as disease management programs or chronic care programs. The first lever is patient-focused whereas the second physician-focused, yet both can be supported digitally. Additionally, COPD patients’ support needs should be further analyzed and focused on in future research, especially in the context of digital support tools. Future policy changes in Switzerland and elswhere should strengthen the incentive structures for physicians and COPD patients to use disease management programs and to finance digital health interventions.

## Methods

### Data

We used claims data from Groupe Mutuel, a Swiss health insurance with a 10% market share^32^, from 2015 to 2020.

The dataset included basic claims data, contract information, and billing data for each insured person (Table 3). Billing data covered the treatments and prescriptions invoiced during the observation period. For each invoice, the date of treatment/prescription, the amount of the treatment/prescription in CHF, the share covered by the insurance, the tariff at which the treatment/prescription was billed, and the tariff code were available. In case of inpatient treatments billed under the SwissDRG tariff, the DRG code, the length of stay, and the reason for the stay (illness, accident, or pregnancy) were included.

**Table 3:** Overview of data types and variables included in dataset.

Data type	Variable
Basic claims data	Year of birth
	Nationality (Swiss vs non-Swiss)
Contract information data	Type of insurance
	Start (and if available) end date of contract
	Deductible level
	Premium reduction (only if received)
Billing data (per treatment/ prescription)	Date
	Amount in CHF
	Portion covered by insurance
	Applicable tariff (TARMED, TARPSY, SwissDRG)
	Tariff code
	*Only for inpatient stays billed at SwissDRG:* Length of hospital stay, Reason for stay (illness, accident, or pregnancy)
	*For medication:* Pharma code (including package size and daily recommended dosis)

### Sample

We included 13,557 insurees with prescriped COPD medication in our sample. In Switzerland, health insurances are not allowed to save data on diagnoses. Thus, to identify COPD patients in our dataset, we defined the following three inclusion criteria:

**Fig. 3:**
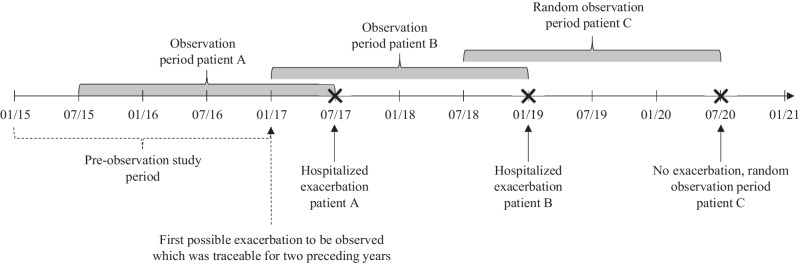
Schematic representation of the study design. The period between 01/15 and 12/16 functioned as a pre-observation period. In case COPD patients underwent a hospitalization due to an exacerbation during this period, these hospitalizations were controlled for in the analysis of the impact of medication adherence on hospitalizations. However, hospitalizations due to exacerbations in the pre-observation period were not treated as observations but only as a control variable. The first possible hospitalization due to an exacerbation to be observed in the sample stated 01/17, where patients’ medication adherence could be tracked for the two preceding years in the pre-observation period. The point in time of the hospitalized exacerbation marked the cut-off date and determined the two years of observation prior to the hospitalized exacerbation (e.g., observation period patients A and B). For COPD patients without a hospitalized exacerbation, a random date marked the cut-off date and the two preceding years were used for the observation (e.g., random observation period patient C).

- Therapy with long-acting medication specific for chronic pulmonary diseases: long-acting medication should be taken on a regular basis, but the medication or rather the active component itself might have changed over time depending on the COPD patient’s health status. Therefore, all medications defined as long-acting medications in the GOLD standard^2^ were grouped together to identify COPD patients: Long-acting beta2 agonists (LABA), long-acting antimuscarinic antagonists (LAMA), the combination of LABA and LAMA, the combination of LABA and corticosteroid (ICS), the combination of LAMA and ICS, and the triple combination ofLABA, LAMA, and ICS. All COPD patients who received any of these medications at least once during the observation period were included.
- Age (>40 years) to distinguish between COPD and asthma patients: long-acting medication can be prescribed to asthma as well as to COPD patients. The literature suggests that patients older than 40 years are more likely to suffer from COPD than from asthma if they take at least one of the above-mentioned medications^2^. Thus, to exclude asthma patients, we only included insured persons older than 40 years in our sample.
- Availability of claims data for at least two consecutive years prior to the exacerbation: a hospitalized exacerbation was identified with the base DRG E65 (COPD). The date of the hospitalized exacerbation was the cut-off date and the observation of a COPD patient started two years before (Fig. 1). Thereby, 2015 and 2016 served as a pre-observation period, as exacerbations between 1 January 2015 and 31 December 2016 were not traceable for two years within our dataset. Additionally, for COPD patients with multiple hospitalized exacerbations, only the first hospitalized exacerbation, which allows an ex-ante observation period of two years (i.e., from 01/2017 onwards), was used to ensure independent observations. For COPD patients without hospitalized exacerbations, by definition, there is no exacerbation day that could have been used as a cut-off date. Therefore, we chose a random period of two years. This ensured independent observations even for non-hospitalized COPD patients.

### Statistical Model

Using real world data, we approximated COPD patient’s medication adherence by calculating their PDC. The PDC represents the proportion of days covered by COPD patients’ personal home-inventory of long-acting medication taken with inhalers during the period of investigation^21^. To estimate a patient’s medication adherence and its impact on the likelihood of hospitalization due to exacerbations, we applied the following calculations:

Patient Medication Reserve: We calculated a patient’s remaining supply of a long-acting medication on a specific day. The reserve depended on the previous day’s reserve, the period when the medication was bought last respecting the package size (in number of doses), and the recommended daily dose. If a patient switched between different medication categories (e.g., from LABA to LAMA), we assumed both medications were taken until one was no longer purchased.

The proportion of days covered (PDC): We determined the PDC for each COPD patient. We calculated the proportion of days by dividing the number of days with enough medication supply by the total observation period. The observation period in this study was set to 720 years, i.e., two years.

To assess the causal effect of medication adherence on the probability of hospitalized exacerbations, we relied on the causal Rubin effect^33^. We measured the PDC’s Average Treatment Effect (ATE) on hospitalized exacerbations^34^. COPD patients were divided into five categories (C1 = 0 to <20%, C2 = 20% to <40%, C3 = 40% to <60%, C4 = 60% to <80%, and C5 = 80% to <100%) based on their PDC values. ATE is the difference between the expected outcomes (hospitalization) for patients in different adherence categories.

To calculate the effect of medication adherence on hospitalized exacerbations, a logistic regression model was used due to the binary nature of the dependent variable (hospitalization or no hospitalization). In the model, we controlled for socioeconomic factors (such as age, franchise level, chosen franchise increase or decrease, and premium reduction), and medical factors (such as hospitalized exacerbations in the first two years of the observation period, purchase of short-acting medication, purchase of methylxanthine, purchase of phosphodiesterase 4 inhibitors, and purchase of mucolytic). The influence of medication adherence was estimated as a linear combination of the category values (C2 to C5) (for further information see Supplementary Information I).

### Reporting summary

Further information on research design is available in the Nature Research Reporting Summary linked to this article.

### Supplementary information

Supplemental material

Reporting Summary

Working Paper Series

## Data Availability

The data used for this study is available from Groupe Mutuel Foundation but restrictions apply to the availability of data. Data is however available from the authors upon reasonable request and with permission of Groupe Mutuel Foundation. The underlying code for this study is not publicly available but may be made available to qualified researchers on reasonable request from the corresponding author.
