# Supplementary material for: Medication adherence halves COPD patients’ hospitalization risk – evidence from Swiss health insurance data
Source: NPJ Prim Care Respir Med. 2024 Mar 7;34:1. doi: 10.1038/s41533-024-00361-2 (PMC10920735; doi:10.1038/s41533-024-00361-2)
Supplement: Supplementary file 1 — Supplemental material [file 41533_2024_361_MOESM1_ESM.pdf]

Supplementary Information I: Formulas on the calculation of the proportion of days covered (PDC)

To approximate the PDC, the following formula were used:

$$R_{t,LA} = \max\left(R_{t-1,LA} + \mathbb{1}_{\{Medication\ bought\ in\ Period\ t\}}\, S_{LA} - D_{LA}, 0\right) \quad (1)$$

$$MPR = \frac{\sum_{t=1}^{T} \mathbb{1}_{\{R_{t,LA}>0\}}}{T} \quad (2)$$

Formula (1) represents the patient's reserve *R* of a long-acting medication *LA* on a particular day *t* in recursive form. The reserve $R_{t,LA}$ depends on the reserve $R_{t-1,LA}$ of the previous day *(t-1)* and the period when medication was bought last respecting the package size (in the number of doses) S_{LA} and the recommended daily dose D_{LA}. If a COPD patient switched from one medication category to another, such as from LABA to LAMA, we assume both medications are taken simultaneously until one is no longer purchased. With formula (2), we calculate the PDC per COPD patient by dividing the sum of all days with enough medication reserve by the total days of the observation period, i.e., by 720 days.

To estimate the causal effect of medication adherence on the probability of a hospitalized exacerbation, we rely on the causal Rubin effect [21]. Accordingly, we quantify the average treatment effect (ATE) of the PDC on hospitalized exacerbations [22]. As the treatment variable for calculating the ATE must be finite, we divided COPD patients into five quintiles Q_q with $q \in \{1, 2, 3, 4, 5\}$ based on COPD patients' respective PDCs. Each COPD patient has a potential outcome *y* for the individual adherence status *q* denoted by the value of the dummy variable Q_q (i.e., $Q_q = 1$ means "COPD patient belongs to the group with adherence status *q*" and $Q_q = 0$ means "COPD patient does not belong to the group with adherence status *q*"). Formally, we define the ATE as

$$\beta_{\mathrm{ate,q}} = \mathrm{E}\left(\mathrm{y}_q - \mathrm{y}_1\right) \quad (3)$$

with E representing whether a COPD patient was hospitalized for exacerbation. The control group is the first quintile Q_1, which includes all COPD patients with a PDC of 0 to < 20%.

Quintiles for q_2 through q_5 have a PDC of 20% to < 40%, 40% to < 60%, 60% to < 80%, and 80% to < 100%. Thus, the respective ATE is estimated on a total of four levels. The effect measure $\beta_{ate,q}$ indicates the average reduction in the probability of being hospitalized due to an exacerbation (for information on the satisfaction of the "conditional independence assumption" see Further Specification I).

Logistic regression is used due to the binary nature of the dependent variable (hospitalized exacerbation *E = 1*, no hospitalized exacerbation *E = 0*):

$$P\big(E = 1 \mid \beta_0 + \beta_q\, Q'_q + \gamma_x\, X' + \mu_w\, W'\big) = S\big(\beta_0 + \beta_q\, Q'_q + \gamma_x\, X' + \mu_w\, W'\big) \quad (4)$$

with

$$S\big(\beta_0 + \beta_q\, Q'_q + \gamma_x\, X' + \mu_w\, W'\big) = \frac{1}{1 + e^{-(\beta_0 + \beta_q\, Q'_q + \gamma_x\, X' + \mu_w\, W')}} \quad (5)$$

where the influence of medication adherence is estimated by

$$\beta_q\, Q'_q = \beta_2\, Q_2 + \beta_3\, Q_3 + \beta_4\, Q_4 + \beta_5\, Q_5 \quad (6)$$

and the influence of socioeconomic factors is taken into account with

$$\gamma_x\, X' = \gamma_{1f}\, F_f + \gamma_2\, F^+ + \gamma_3\, F^- + \gamma_4\, R + \gamma_5\, A \quad (7)$$

and finally medical factors are considered with

$$\mu_w\, W' = \mu_1\, E_{t-2} + \mu_2 B + \mu_3\, M + \mu_4\, D + \mu_5\, K \quad (8).$$

Simplified, formula (4) expresses the probability P per COPD patient of being hospitalized with an exacerbation (E = 1), depending on the quintile of medication reserve *Qq* (formula (6)), socioeconomic factors *X* (formula (7)), and medical factors *W* (formula (8)). Due to the logistic

nature of the regression analysis, formula (5) shows the exponential form of the regression equation. An overview of the notation of the variables is provided in Further Specification II.

To satisfy identifying assumption (1), i.e., the CIA, variables that have an effect on both the quintile assignment and the dependent variable are used as control variables that we grouped into two categories: Socioeconomic factors (age A, franchise level F_f, chosen franchise increase F^+ or decrease F^-, and premium reduction R) and medical factors (hospitalized exazerbations in the first two years of the observation period E_{t-2}, purchase of short-acting medication B, purchase of methylaxthine M, purchase of phosphodiesterase 4 inhibitors D, and purchase of mucolytic K). The deductible F_f serves as a proxy of risk preference relative to expected healthcare expenditures. Since it is possible to change the deductible rate at the beginning of each calender year, we use the deductible F_f an insured person has at the very end of the observation period. To control for changing deductible rates between two subsequent years, the increase (F^+) and the decrease (F^-) of the deductible is included as a control variable. The deductible F_f and the changes in deductibles (F^+ and F^-) approximate the severity of COPD, as individuals who expect high health care costs to occur in the upcoming year may accordingly adjust their deductible. In addition, the premium reduction R is included to control for the socioeconomic status approximated by income. Only insured with low income are eligible to obtain a premium reduction.

Consequently, whether a COPD patient has ever been hospitalized for an exacerbation during the observation period is taken into account (E_{t-2}). This control variable approaches the COPD patients ability on dealing with the disease. Frequent hospitalized exacerbations may indicate poor disease management. Furthermore, short-acting medications (B) are used as a proxy for COPD severity. The GOLD standard defines two short-acting medication groups, namely beta$_2$ agonists (SABA) and antimuscarinic antagonists (SAMA), and their combination [2]. These

medications may be taken in addition to the long-acting medications and indicate that this COPD patient has higher disease severity. Other agents used in the treatment of COPD include methylxanthines (*M*), phosphodiesterase-4 inhibitors (*D*), and mucolytics (*K*). These are used during acute worsening of lung function and hint at a non-hospitalized exacerbation.

Further Specification I: Conditional independence assumption

The most important difference between the identification of the ATE in an experimental setting, e.g., in a randomized controlled trial, versus the "Selection on Observables" applied in this study, is a weaker form of independence *assumption*, i.e., a *conditional independence assumption* (CIA). The CIA is violated when unobserved or empirically untested or untestable influencing factors are present. In such cases, it is assumed that most likely unexplained confounding factors affect the ATE.

According to these considerations, an empirical strategy suitable for the "Selection on Observables" method is used. Therefore, five identifying assumptions for each patient in this sample apply: 1) The assignment of a case to the treatment and control group according to CIA depends on the characteristics of the control variables. Hence, the treatment must actually depend on the values of the observed control variables measured for the case. This means that all necessary control variables are observed. 2) Each patient (i.e., each observation) could receive or not receive the treatment. This implies that there should be no patient subgroups (e.g., patients above 80 years) that occur in only one of the quintiles. By using the descriptive results, violations of this assumption can be excluded. 3) The treatment does not affect the control variables in a way that is related to the outcome. This means that e.g., that age potentially affects medication reserve, but medication reserve does not affect patient age. 4) A patient's treatment status only affects the individual outcome. This signalizes that a patient's own medication reserve

does not affect another patient's likelihood of a hospitalized exacerbation. 5) There are only as many different potential outcomes as there are different treatments and one is observed for each subject. This indicates that the variation in medication reserve among multiple patients within a quintile should be small.

Further Specification II: List of model variables with corresponding abbreviations

Variable/ Category	Abbreviation	Description	Variable type
Probability function	P	The likelihood that a patient will be hospitalized due to an exacerbation	Percentage value between 0 and 100
Exponential function	S	Designation of the exponential function of a logistic regression	-
Dependent variable	E	Hospitalized exacerbation	Binary, 0 or 1
Independent variable of interest	Q_q	Quintile q of the proportion of days with medication reserve in all days in the observation period.	Binary, 0 or 1 (per quintile)
Socioeconomic control variables	A	Age	Steady
	F_f	Most recent deductible (with f equal to 350, 500, 1,000, 1,500, 2,000, or 2,500), i.e., at the end of the observation period	Binary, 0 or 1 (per franchise)
	F^+	Increase in deductible during the observation period	Binary, 0 or 1
	F^-	Reduction of the deductible during the observation period	Binary, 0 or 1
	R	Premium reduction	Binary, 0 or 1
Medical control variables	E_{t-2}	Hospitalized exacerbations in the two years of the observation period	Binary, 0 or 1
	B	Purchase of short-acting medication	Binary, 0 or 1
	M	Purchase of methylxanthine	Binary, 0 or 1
	D	Purchase of phosphodiesterase 4 inhibitors	Binary, 0 or 1
	K	Purchase of mucolytic	Binary, 0 or 1
